# Synthesis and In Vitro Evaluation of Aspartic Acid Based Microgels for Sustained Drug Delivery

**DOI:** 10.3390/gels8010012

**Published:** 2021-12-24

**Authors:** Muhammad Suhail, An Xie, Jia-Yu Liu, Wan-Chu Hsieh, Yu-Wen Lin, Muhammad Usman Minhas, Pao-Chu Wu

**Affiliations:** 1 School of Pharmacy, Kaohsiung Medical University, 100 Shih-Chuan 1st Road, Kaohsiung 80708, Taiwan; Suhailpharmacist26@gmail.com (M.S.); andrewxie1971@gmail.com (A.X.); u109830006@kmu.edu.tw (J.-Y.L.); wanchuhsieh@gmail.com (W.-C.H.); u108530006@kmu.edu.tw (Y.-W.L.); 2 College of Pharmacy, University of Sargodha, Sargodha 40100, Pakistan; 3 Department of Medical Research, Kaohsiung Medical University Hospital, Kaohsiung 80708, Taiwan; 4 Drug Development and Value Creation Research Center, Kaohsiung Medical University, Kaohsiung 80708, Taiwan

**Keywords:** microgels, drug loading, dynamic swelling, percent drug release

## Abstract

The main focus of the current study was to sustain the releasing behavior of theophylline by fabricated polymeric microgels. The free radical polymerization technique was used for the development of aspartic acid-co-poly(2-acrylamido-2-methylpropanesulfonic acid) microgels while using various combinations of aspartic acid, 2-acrylamido-2-methylpropanesulfonic acid, and N′,N′-methylene bisacrylamide as a polymer, monomer, and cross-linker, respectively. Ammonium peroxodisulfate and sodium hydrogen sulfite were used as initiators. Characterizations such as DSC, TGA, SEM, FTIR, and PXRD were performed for the fabricated microgels to assess their thermal stability with unreacted polymer and monomer, their surface morphology, the formation of a new polymeric system of microgels by evaluating the cross-linking of functional groups of the microgels’ contents, and to analyze the reduction in crystallinity of the theophylline by fabricated microgels. Various studies such as dynamic swelling, drug loading, sol–gel analysis, in vitro drug release studies, and kinetic modeling were carried out for the developed microgels. Both dynamic swelling and percent drug release were found higher at pH 7.4 as compared to pH 1.2 due to the deprotonation of functional groups of aspartic acid and AMPS. Similarly, sol–gel analysis was performed and an increase in gel fraction was observed with the increasing concentration of microgel contents, while sol fraction was decreased. Conclusively, the prepared carrier system has the potential to sustain the release of the theophylline for an extended period of time.

## 1. Introduction

Microgels are solvent-swollen, hydrogel, micro-particulate systems possessing discrete particles within the range of 20 nm to 50 µm. Microgels are widely recognized as a promising material for drug delivery [1]. The benefits of microgels are their synthesis through a simple procedure and the control over key features including size and functionality, key for regulating the precise binding of drugs, release kinetics, high stability and shelf-life, biodistribution and specific delivery, biocompatibility, bioaccumulation, degradation, and functionality in the context of a drug delivery application. Due to their high potential in biomedicine and drug delivery, great attention has been placed on microgels, especially in academic research. A number of advantages are presented by microgels over other nanoparticle-based vehicles in terms of in vivo application. The main benefit of microgels is their straightforward preparation procedure, which results in highly monodisperse nanoparticles in most cases. The resulting water-swollen, polymeric network of microgels is highly hydrophilic in nature and a low interfacial energy is presented in a biological environment, decreasing the nonspecific interactions with proteins (opsonization) and enhancing their biocompatibility and bioavailability [2,3].

**Figure 1:**
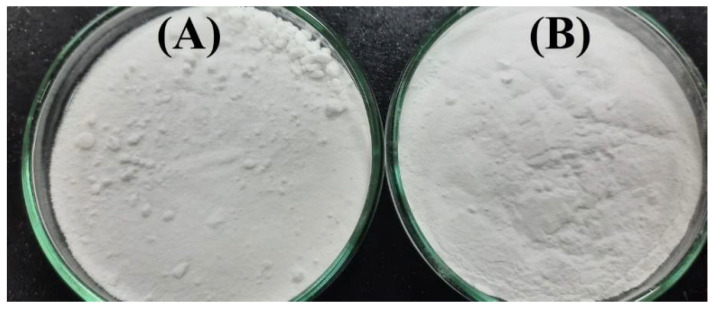
Physical appearance of formulations with increasing concentration of (**A**) MBA, and (**B**) ASPA/AMPS of ASPA-pAMPS microgels.

Poly(aspartic acid) (ASPA), a synthetic polymer, is composed of free carboxylic groups or amino groups based on natural amino acid, as shown in Figure 1 [4]. Its aqueous solubility, biodegradability, and non-toxic nature are the main characteristics that enabled ASPA as a suitable candidate for drug delivery [5]. Similar to other ionic polymers whose swelling index is enhanced by either lowering the ionic strength or increasing the pH of the medium, ASPA shows its response to ionic strength and to the pH of the medium due to the presence of carboxylic groups. The ionization of carboxylic groups leads to a polyelectrolyte effect [6,7]. Negative charges are produced throughout the network due to ionization or deprotonation that results in the confirmation of the extended chain and a globule to coil transition [8]. 2-Acrylamido-2-methylpropanesulfonic acid (AMPS) is a hydrophilic monomer. It is ionic and non-ionic in nature, having a pKa value of 2. AMPS is a white crystalline powder that dissolves in water rapidly due to its hydrophilic nature, but its solubility is limited to polar organic solvents. The swellability of AMPS is highly dependent on ionized sulfonate groups. Due to the presence of sulfonic functional groups, AMPS shows a better stability against hydrolysis and a strong resistance to salt. AMPS has the capability to highly swell once exposed to a particular pH of the medium. The swelling of AMPS depends upon the polymer used in combination with them. It plays an important role in drug delivery systems. Malik et al. and coworkers prepared chitosan/xanthan gum-based hydrogels wherein AMPS was used as a monomer and demonstrated that the developed network of hydrogels released the antiviral drug acyclovir in a controlled way [9]. Similarly, Abid et al. (2021) developed xanthan gum and polyvinyl pyrolidone-co-poly(AMPS) hydrogels and reported the controlled delivery of venlafaxine [10].

Theophylline (TP) is an alkaloid and commonly used as a bronchodilator drug in the treatment of chronic obstructive pulmonary disease [11,12]. TP is obtained from *Camellia sinensis* leaves. When TP is administered, the bronchioles and muscles of pulmonary sanguine vessels are directly relaxed, which demonstrates the relaxing and bronchodilator effect of TP upon the smooth muscles [13]. The half-life of TP is in the range of 6–12 h, but commonly reported as 8 h, whereas in smoking patients, the half-life is reduced to 5 h. In order to avoid large fluctuations of the plasma concentration, TP needs to be administered three to four times in a day [14]. However, taking TP several times in a day leads to severe complications such as nausea, vomiting, insomnia, abdominal pain, jitteriness, and a rapid or irregular heartbeat, which results in the high release of medication. It also reduces patient compliance [15]. Therefore, to overwhelm all these complications and improve the patient compliance, a polymeric system is required to sustain the TP’s release. Zhang and coworkers prepared TP-loaded microspheres of chitosan/β-cyclodextrin by the spray drying method and reported the sustained release of TP for 6 h at pH 6.8 [16]. Similarly, Ahirrao et al. (2014) developed hydrogel beads of sodium alginate and reported the maximum sustained release of TP for 11 h [17]. However, a lot of work is still needed in order to overcome the challenges faced by TP, especially due to its frequent administration. Therefore, the authors have prepared aspartic acid-based microgels for the sustained release of TP for 24 h.

The literature reveals that microgel is one of the most suitable carrier systems for the sustained/controlled release of drugs. Hence, different researchers have formulated microgels for the sustained/controlled delivery of drugs such as poly(N-isopropylacrylamide) microgels, which were developed for the sustained release of naltrexone for up to 5 h by Kjøniksen and coworkers [18]. Similarly, Babu et al. (2006) prepared pH-sensitive microgels of sodium alginate/acrylic acid and demonstrated the controlled release of ibuprofen up to 12 h [19]. Comparing the release behavior of the currently fabricated microgels with the previously reported data, the developed microgels can be considered most suitable for the sustained release of drugs. The novelty of prepared microgels can be connected with the cross-linking of ASPA with AMPS by N′,N′-methylene bisacrylamide (MBA) in the presence of initiators. Due to its unique features such as good biodegradability, solubility in water, and a non-toxic nature, ASPA has recently gained much attention, especially regarding drug delivery systems. Due to its pH-sensitive nature, the use of ASPA has been increased, especially in the development of pH-sensitive drug carrier systems such as hydrogels, microgels, and nanogels, etc. The pH sensitivity of ASPA is increased with the increase in pH of the medium due to the presence of carboxylate groups, which enable the ASPA to deprotonate at high pH values. Similarly, AMPS is hydrophilic in nature, and thus used widely in the preparation of different pharmaceutical products. As a good hydrophilic monomer, the introduction of AMPS into a polymer network increases the pH sensitivity, swelling ratio, and drug release of the developed drug carrier system. Hence, the recent combination of ASPA and AMPS has enabled the polymeric microgel to highly swell at high pH values due to its pH-sensitive contents and, as a result, maximum swelling and drug loading are observed. Similarly, a high drug release of the fabricated microgel at high pH values protects the stomach from the side effects of the drug and also the drug itself from stomach acidity. Hence, we can conclude that fabricated microgels could be considered as an ideal drug carrier system for the sustained release of theophylline and of other drugs too.

Here, we report the synthesis of aspartic acid-based microgels for TP sustained release. Different concentrations of the polymer ASPA, the monomer AMPS, and the cross-linker MBA (N′,N′-methylene bisacrylamide) were employed in the presence of the initiator APS (ammonium peroxodisulfate) and SHS (sodium hydrogen sulfite) for the fabrication of aspartic acid-co-poly(2-acrylamido-2-methylpropanesulfonic acid) microgels to sustain the release of TP for a prolonged period of time. Various studies, such as dynamic swelling, drug loading, sol–gel fraction, in vitro studies, and kinetic modeling were carried out. Similarly, characterizations such as DSC, TGA, SEM, FTIR, and PXRD were conducted to know and assess the different aspects of the developed microgels.

## 2. Results and Discussion

### 2.1. Physical Appearance

The physical appearance of the fabricated microgels was white in color, as shown in Figure 1A,B. The difference was in the hardness of the formulation. All the formulations of MBA (Figure 1A) with an increasing concentration were hard and dense. The bulk density increased while the pore size decreased. The formulations of ASPA and AMPS (Figure 1B) with an increasing concentration were porous with less bulk density.

### 2.2. Dynamic Swelling

**Figure 2:**
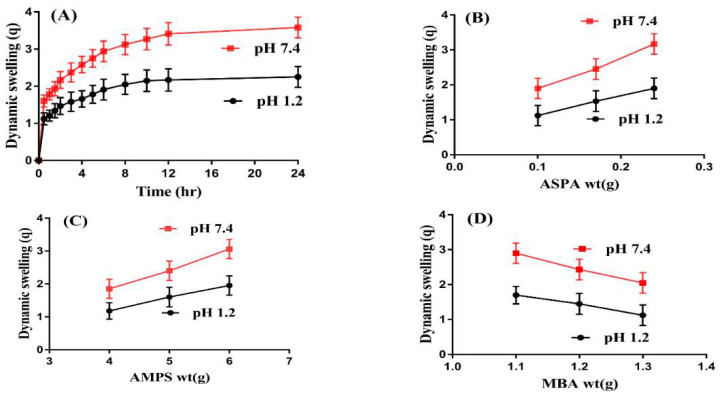
Effects of (**A**) pH, (**B**) ASPA, (**C**) AMPS, and (**D**) MBA on dynamic swelling of ASPA-pAMPS microgels.

Swelling studies were carried out for ASPA-pAMPS microgels to determine the swelling index of the microgels at two different pH media, i.e., pH 1.2 and 7.4, respectively, as shown in Figure 2A–D. Higher swelling at both pHs was exhibited for developed microgels but, due to the presence of COOH and NH groups of ASPA, swelling at pH 7.4 was observed higher than pH 1.2 (Figure 2A). COOH and NH functional groups of ASPA were protonated at a lower pH of 1.2 and formed a conjugate with counter ions via strong hydrogen bonding and, as a result, a low swelling index of the fabricated microgels was observed at pH 1.2. However, with the increase in pH of the medium, deprotonation of COOH and NH groups occurred, which leads to an increase in charge density and generates strong electrostatic repulsive forces. These electrostatic repulsive forces result in higher expansion/swelling of microgels due to the high charge density of the same functional groups, which repel each other, and, as a result, maximum swelling is observed. Hence, as the pH of the medium is enhanced, the swelling of the fabricated microgels is increased in the same pattern, and vice versa [20]. Similarly, SO_3_H groups of AMSP were protonated at pH 1.2 because the pKa value of SO_3_H group was almost 1.9. Due to the protonation of SO_3_H groups, the charge density of SO_3_H groups was decreased due to the formation of a conjugate with counter ions by strong hydrogen bonding, and hence a decrease in swelling was observed. On the other hand, SO_3_H groups of AMPS were deprotonated at a high pH of 7.4, which leads to high charge density and, as a result, strong electrostatic repulsive forces are produced, which repel each other, and maximum swelling is achieved [9,21,22].

ASPA, AMPS, and MBA also influence the dynamic swelling of ASPA-pAMPS microgels at both pH values, as shown in Figure 2B–D. The swelling increased as the concentration of the ASPA increased (Figure 2B). ASPA has COOH and NH functional groups, and an increase in the concentration of ASPA led to an increase in COOH and NH groups; due to this, charge density is increased and swelling increases. Similarly, a rise was seen in the dynamic swelling of microgels as the concentration of AMPS increased (Figure 2C). AMPS contains SO_3_H groups, and as the concentration of AMPS increased, the generation of SO_3_H groups also increased; due to this, charge density is enhanced and swelling increases, and vice versa [23,24]. Both ionic and non-ionic groups are present in AMPS. As the concentration of ionic groups is increased, the swelling and superabsorbancy capabilities of AMPS-based hydrogels are increased and hence started to dissociate at different pHs [25]. Unlike ASPA and AMPS, a decrease in dynamic swelling was observed as the concentration of the MBA increased (Figure 2D). The bulk density of polymeric microgels is increased with the enhancement of the MBA concentration; due to this, the penetration of water into a microgel network decreases and, as a result, a decline is observed in the dynamic swelling of microgels [26,27].

### 2.3. Drug Loading

Swelling plays an important role in drug loading. The maximum amount of drug will be loaded by microgels if the swelling of the system is high because the larger the pore size, the greater the amount of fluid that will penetrate through the pores into the microgels’ network. Due to this, the dynamic swelling will be greater and, as a result, the maximum amount of drug will be loaded by the microgels, and vice versa [28]. Moreover, the % drug loading was carried out for all formulations of ASPA-pAMPS microgels, as shown in Table 1. The % drug loading increased as the concentration of the ASPA and AMPS increased because the swelling of microgels was enhanced with the increase in the concentration of the ASPA and AMPS [29]. Contrary to ASPA and AMPS, the % drug loading was reduced as the concentration of the MBA increased. The bulk density was increased, due to which, water penetration into microgels decreases, which leads to a reduction in swelling; hence, % drug loading is decreased [28].

### 2.4. Sol–Gel Analysis

Sol–gel analysis was carried out for the developed microgels to know the soluble un-cross-linked and insoluble cross-linked parts of microgels. The gel fraction was increased (Table 1) as the concentration of all microgel contents increased, i.e., ASPA, AMPS, and MBA, respectively. As the concentration of the ASPA increased, the gel fraction was increased because a high amount of free radicals were generated by ASPA for the monomer contents, which led to a fast polymerization process among the microgels’ contents, therefore the gel fraction increased. Samanta et al. (2014) also reported that, as the concentration of polymer increases, the polymerization process among the hydrogels’ contents is enhanced, which leads to greater gelation [30]. Similarly, a greater amount of SO_3_H groups were produced as the concentration of AMPS increased. The higher the SO_3_H groups, the faster the chemical reaction would be between polymer and monomer on their respective reactive sites and thus higher the gel fraction, and vice versa. The gel fraction is increased up to certain limit because the gel fraction is decreased if a very high concentration of AMPS is used. In such a condition, the AMPS contents already occupied the available reactive sites of the polymer, any further increase leads to steric hindrance effects and to the formation of a layer on the backbone of the polymer, due to which the pore size of the system decreases, the hardness of the system increases and, as a result, the gel fraction decreases [31]. Similar to ASPA and AMPS, the gel fraction is increased as the concentration of the MBA is increased. The higher the MBA concentration, the faster the cross-linking among the microgel contents will be and the greater the gel fraction [32,33]. Unlike the gel fraction, the sol fraction is decreased with the increase in the concentration of ASPA, AMPS, and MBA because sol fraction is inversely proportional to gel fraction [34], and vice versa.

### 2.5. In Vitro Drug Release Study

**Figure 3:**
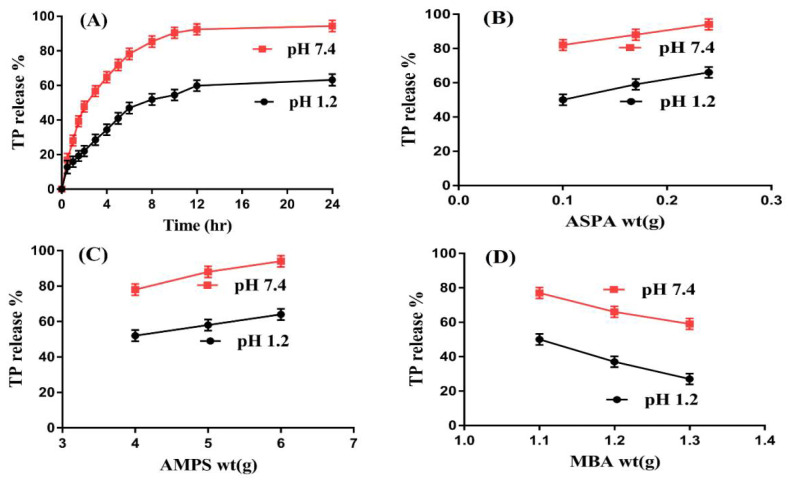

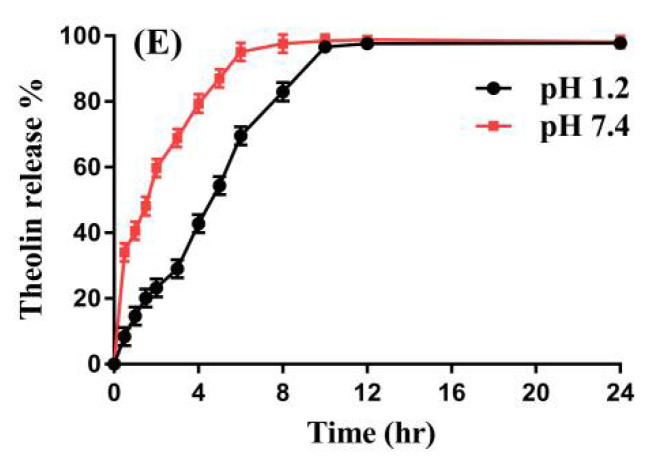
Effect of (**A**) pH, (**B**) ASPA, (**C**) AMPS, and (**D**) MBA on TP percentage release from ASPA-pAMPS microgels, (**E**) effect of pH on drug release from Theolin tablet.

An in vitro drug release study was conducted for fabricated microgels to evaluate the percent drug release from the ASPA-pAMPS microgels at both acidic and basic media, i.e., pHs 1.2 and 7.4, respectively, as indicated in Figure 3A–D. A higher percent drug release (90%) was seen at pH 7.4 as compared to pH 1.2 (60%) (Figure 3A) due to the deprotonation of the functional groups of the polymer and monomer. ASPA contains COOH and NH groups. Therefore, the functional groups of ASPA were protonated at a lower pH of 1.2 and formed a conjugate with other counter ions. Strong hydrogen bonding occurred, due to which, swelling and percent drug release were observed as almost low at pH 1.2. However, as the pH increased from 1.2 to 7.4, the deprotonation of COOH and NH groups of the ASPA occurred, which led to higher charge density and, as a result, strong electrostatic repulsive forces were generated. The same charges repelled each other, due to which, greater swelling and percent drug release were ultimately detected. Similarly, AMPS contained SO_3_H groups, which resulted in an increase in swelling and percent drug release at a higher pH of 7.4 due to the deprotonation of SO_3_H groups [9,35]. Similarly, an in vitro drug release study was carried out for the commercially available tablets Theolin S.R (250 mg, PeiLi Pharmaceutical IND. Co., Ltd, Taichung, Taiwan) at both pH 1.2 and 7.4, respectively, as shown in Figure 3E. A drug release of 96% from Theolin was observed for an initial 10 h at pH 1.2, whereas at pH 7.4, a drug release of 96–98% was detected for an initial 6–8 h. Comparing the percent drug release from the commercial product and the fabricated microgels, we can see that the developed system significantly sustained the release of TP for an extended period of time.

Microgel contents, i.e., ASPA, AMPS, and MBA, also influenced the percent drug release from the developed microgels at both pH 1.2 and 7.4, respectively. The percent drug release increased with an increase in the concentration of ASPA (Figure 3B) because the increase in the generation of COOH and NH groups occurred with the increase in ASPA concentration, and vice versa. Similarly, an increase in percent drug release was observed as the AMPS concentration was increased (Figure 3C). The possible reason for this is the generation of maximum SO_3_H functional groups, which leads to higher swelling and percent drug release. Contrary to ASPA and AMPS, a decrease was seen in percent drug release with an increase in the concentration of MBA (Figure 3D). The possible reason is the higher bulk density, which leads to a reduction in swelling and percent drug release [36]. Ali et al. (2014) developed polyvinyl alcohol-based hydrogels and reported that the percent drug release increased as the concentration of polymer and monomer increased, while it decreased with the increase in the concentration of the cross-linker [37].

Kinetic models such as zero order, first order, Higuchi, and Korsmeyer–Peppas models were carried out for all formulations of ASPA-pAMPS microgels to deduce the release mechanism of the drug from the fabricated microgels. The “r” values represent the regression co-efficient. The results of the kinetic models showed that all formulations exhibited the first order of the kinetic model because the “r” values of first order were greater than the “r” values of the respective models, as shown in Table 2 [38]. The “n” values determine the type of diffusion, i.e., Fickian diffusion/non-Fickian diffusion. If the “n” value is greater than 0.45, it indicates that the diffusion is non-Fickian, while if the “n” value is equal to or less than 0.45, it means that the diffusion is Fickian, and vice versa. The “n” values for all formulations were found within the range of 0.4886—0.8526, which indicated non-Fickian diffusion.

### 2.6. DSC Analysis

**Figure 4:**
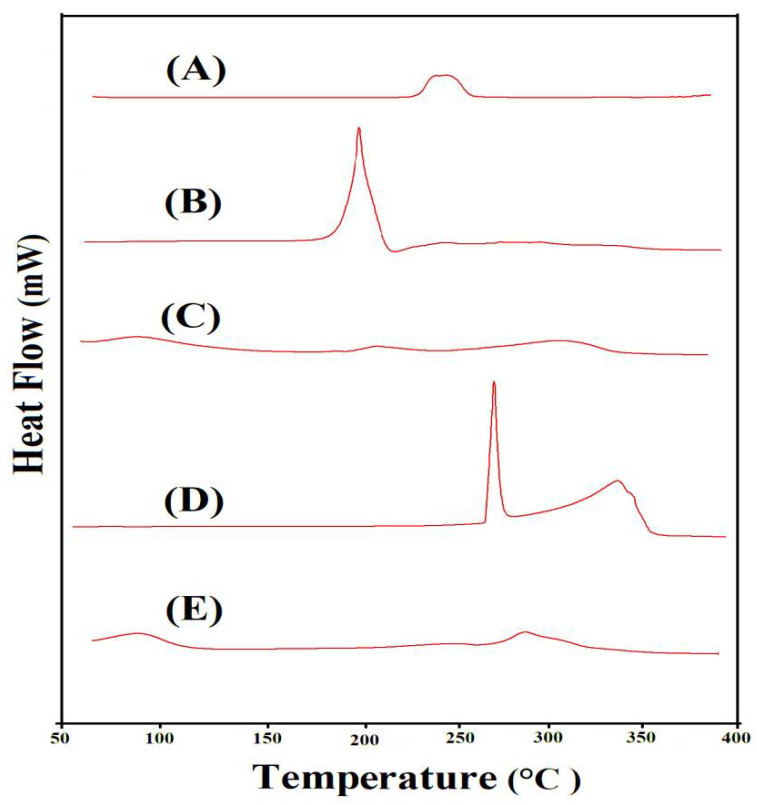
DSC of (A) ASPA, (B) AMPS, (C) ASPA-pAMPS microgels (AFn-3), (D) TP, and (E) loaded ASPA-pAMPS microgels (AFn-3).

To investigate the thermal stability of ASPA, AMPS, unloaded ASPA-pAMPS microgels, TP, and drug-loaded ASPA-pAMPS microgels, DSC was conducted, as shown in Figure 4A–E. The DSC of ASPA (Figure 4A) revealed an endothermic peak at 245 °C concerned with moisture loss, whereas an exothermic was observed at 255 °C, which demonstrates the ASPA’s degradation [39]. The DSC of AMPS (Figure 4B) indicated dehydration by an endothermic peak at 183 °C, while the glass transition temperature was revealed by an exothermic peak at 170 °C. Moreover, the degradation of AMPS was assigned by an exothermic peak at 202 °C [40]. Similarly, the DSC of ASPA-pAMPS microgels (Figure 4C) indicated two endothermic peaks at 205 °C and 310 °C. The endothermic peak of the polymer was moved from 245 °C to 310 °C in ASPA-pAMPS microgels, which indicated the high stability of the developed microgels. We can conclude from the above discussion that the developed microgels’ network is more thermally stable than its basic contents, i.e., ASPA and AMPS. This all means that ASPA, AMPS, and MBA successfully polymerized and fabricated a suitable and stable microgel network for a sustained drug delivery system. Khan et al. (2020) reported the same results as our study, which further supports our hypothesis [41]. The DSC of TP (Figure 4D) revealed an endothermic peak at 272 °C, whereas two broad exothermic peaks were shown at 260 and 342 °C, respectively. The broad exothermic peak of TP at 260 °C could be seen in the DSC of the loaded ASPA-pAMPS microgels (Figure 4E) at 290 °C. The slight modification in the peak position of TP was due to their loading by the developed microgels, which revealed no interaction of TP with the microgels’ contents.

### 2.7. TGA Analysis

**Figure 5:**
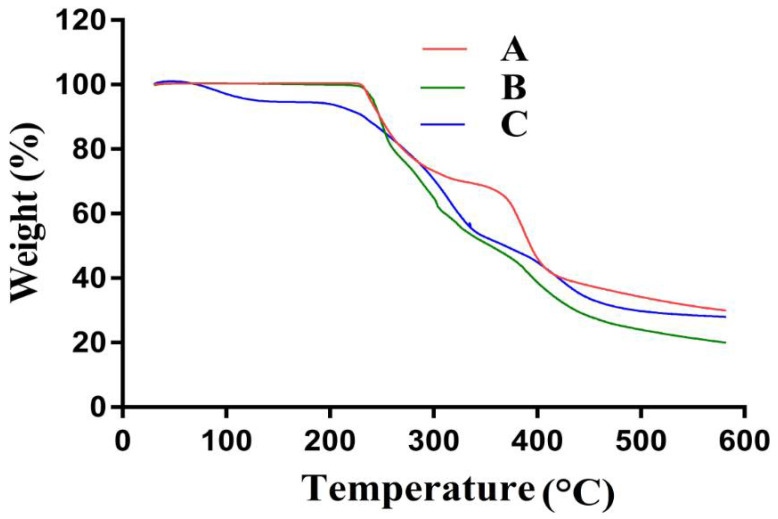
TGA of (A) ASPA, (B) AMPS, and (C) ASPA-pAMPS microgels (AFn-3).

TGA was conducted to evaluate and analyze the thermal stability of ASPA, AMPS, and ASPA-pAMPS microgels, as shown in Figure 5A–C. The TGA of ASPA (Figure 5A) revealed a 27% loss in weight until the temperature approached 305 °C due to the loss of surface moisture. As the temperature increased up to 385 °C, a further loss of 13% in weight was perceived. A further increase in temperature led to a rapid decline in weight of ASPA, the degradation of ASPA started at 410 °C, and a further 15% weight loss was detected as the temperature approached 600 °C due to the degradation of the amino and carboxyl groups [42]. The TGA of AMPS (Figure 5B) indicated a weight loss of 7% as the temperature reached 208 °C; a further weight loss of 45% was seen within the temperature range of 210 to 230 °C, which represents the dehydration of AMPS. Similarly, the sulfonic acid group started to decompose at 320 °C and a weight loss of 20% was detected within the temperature range of 230–320 °C. Weight loss continued, and a 22% reduction in weight was seen as the temperature approached 600 °C [22]. The TGA of ASPA-pAMPS microgels is shown in Figure 5C, which shows that the degradation half-life of the developed microgels was t1/2 = 350 °C, thus indicating that the developed polymeric network of microgels has the potential to remain stable at high temperature. A weight loss of 10% was indicated within the temperature range of 100–190 °C, followed by a further weight loss of 55% within the temperature range of 200–450 °C due to a breakdown of COOH and SO_3_H groups of ASPA and AMPS, respectively. Further degradation of the fabricated microgels started at 450 °C and kept going. A further 3% weight loss was perceived by the fabricated microgels until the temperature approached 600 °C. Conclusively, the discussion demonstrates that the fabricated network of microgel was thermally stable due to the cross-linking of its basic unreacted ingredients. B. Singh et al. (2019) developed carbopol-based hydrogels and reported high thermal stability for fabricated hydrogels [43].

### 2.8. Surface Morphology and Particle Size

**Figure 6:**
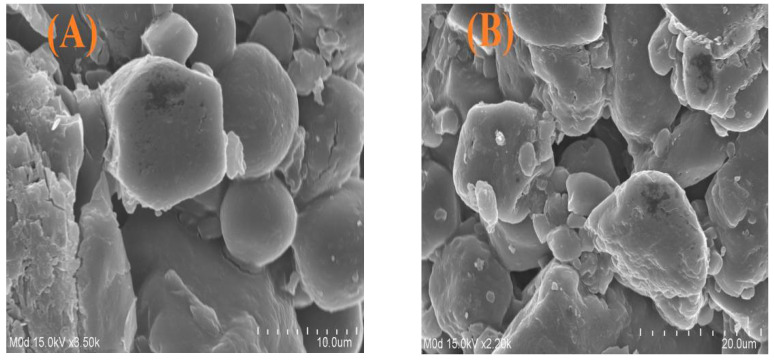

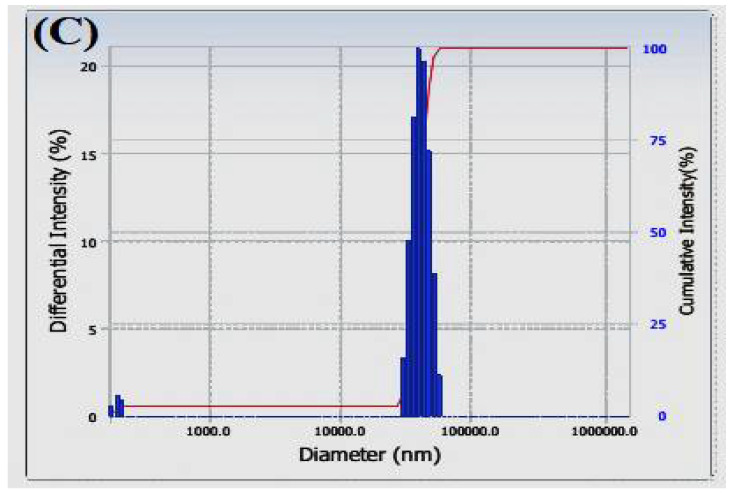
(**A**,**B**) Surface morphology of ASPA-pAMPS microgels (AFn-3), and (**C**) average particle size of ASPA-pAMPS microgels.

SEM was performed at two different magnifications in order to evaluate and analyze the surface morphology of fabricated microgels, as shown in Figure 6A,B. A hard, rough surface with few pores was seen, which demonstrates the successful grafting of the polymer and monomer on their respective sites. The fluid medium penetrated through the pores into the microgels’ network, which results in the swelling of microgels. Hence, the higher the swelling, the greater the drug loading, and therefore the greater the drug release [29], and vice versa. The swelling capability of microgels will be high if their surface is porous and vice versa. The average particle size of ASPA-pAMPS microgels (Figure 6C) was found to be within the range of 26.967 um (26967.4 nm) with a polydispersity index of 0.480, which leads to high swelling, drug loading, and the release of drug [44].

### 2.9. FTIR Analysis

**Figure 7:**
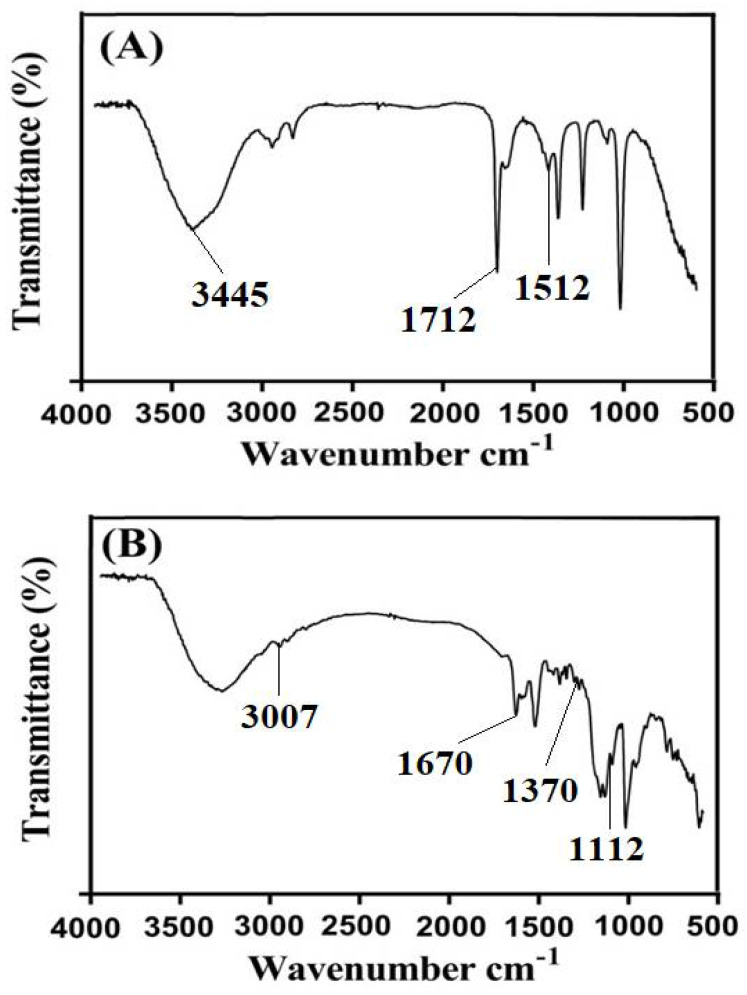

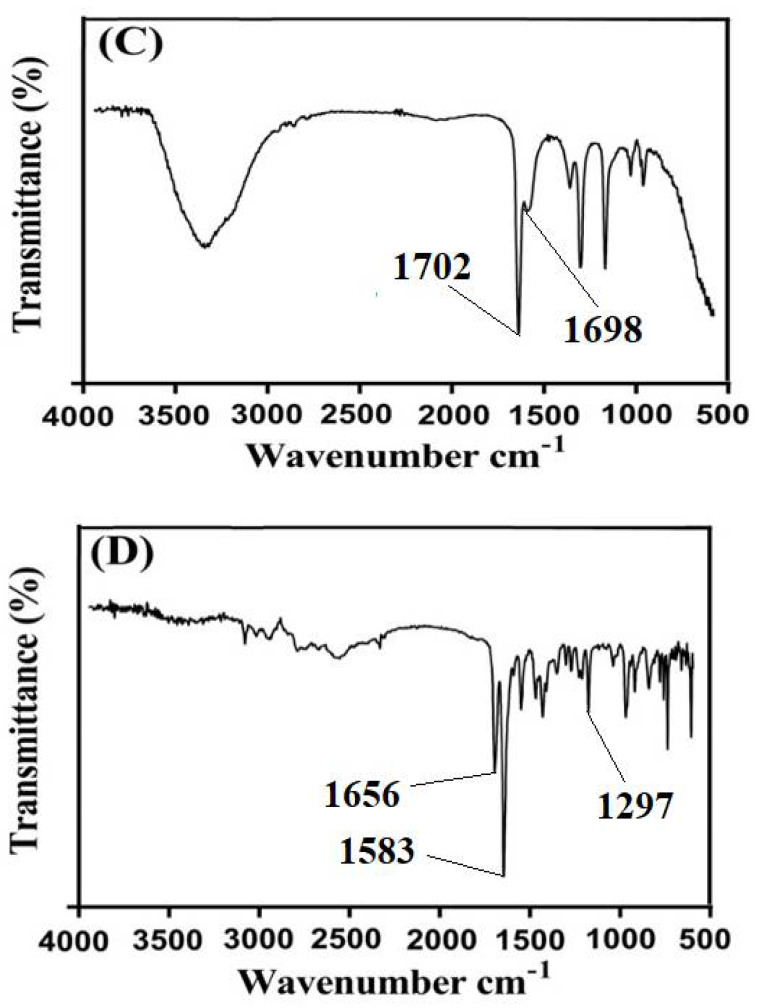

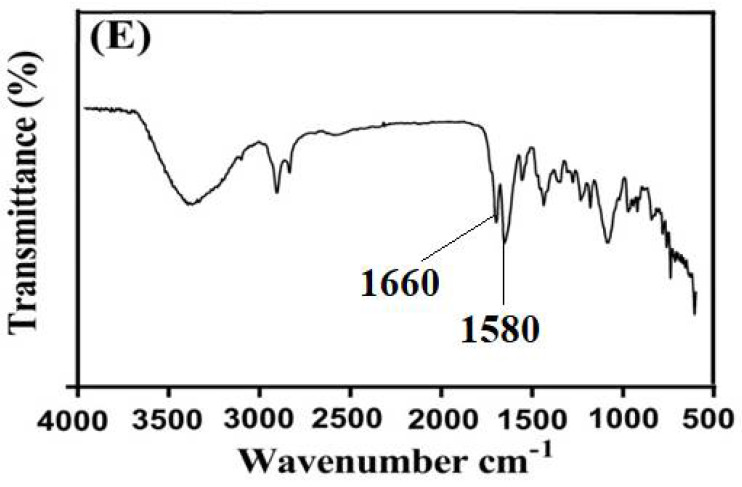
FTIR spectra of (**A**) ASPA, (**B**) AMPS, (**C**) unloaded ASPA-pAMPS microgels (AFn-3), (**D**) TP, and (**E**) loaded ASPA-pAMPS microgels (AFn-3).

FTIR spectra of ASPA, AMPS, unloaded ASPA-pAMPS microgels, TP, and drug-loaded ASPA-pAMPS microgels are shown in Figure 7A–E, respectively. The FTIR spectrum of ASPA is presented in Figure 7A and indicates that the bands at 1562 and 1512 cm^−1^ correspond to N–H of amide. The absorption peak of C=O of the -COOH functional group was observed by a peak at 1712 cm^−1^. Similarly, a broad band at 3445 cm^−1^ indicated the stretching vibration of N–H of the ASPA. The symmetric stretching vibration of carboxylate and OH groups was assigned by peaks at 1413 and 2860–3310 cm^−1^. Zhao et al. (2006) reported the same spectra of ASPA as presented in our current studies, which further supports our observation [4]. Figure 7B indicates the FTIR spectrum of AMPS. C–H stretching of the methyl group of AMPS was assigned by a sharp band at 3007 cm^−1^. The stretching and bending of C=O and N–H groups were assigned by absorption bands at 1670 and 1625 cm^−1^. Similarly, the symmetric and asymmetric stretching vibration of S=O group was indicated by absorption bands at 1112 and 1370 cm^−1^ [45]. The ASPA peak at 1712 cm^−1^ and the AMPS peak at 1670 cm^−1^ shifted to 1702 and 1698 cm^−1^ in ASPA-pAMPS microgels (Figure 7C), while some peaks disappeared. The shifting, disappearance, and formation of new bands revealed the overlapping of AMPS on the backbone of ASPA and the fabrication of ASPA-pAMPS microgels. The FTIR spectra of TP (Figure 7D) assigned prominent bands at 1656, 1583, and 1297 cm^−1^, corresponding to C=O stretching amid, C=C stretching aromatic, and C-O stretching, respectively. The characteristic bands of TP at 1656 and 1583 cm^−1^ shifted slightly to peaks at 1660 and 1580 cm^−1^, respectively, in loaded ASPA-pAMPS microgels (Figure 7E) due to the loading of TP by fabricated microgels. Therefore, no interaction was seen between the TP and microgels’ contents [22].

### 2.10. PXRD Analysis

**Figure 8:**
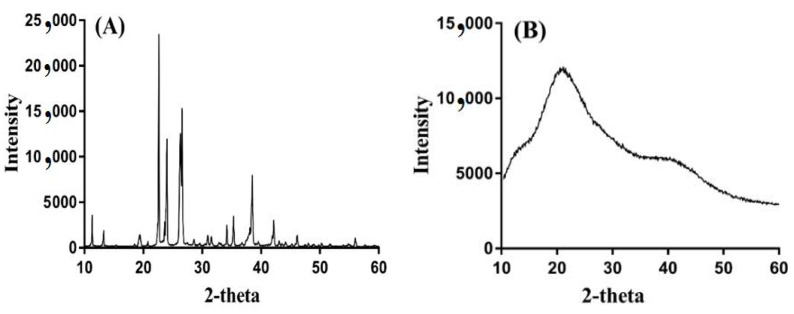

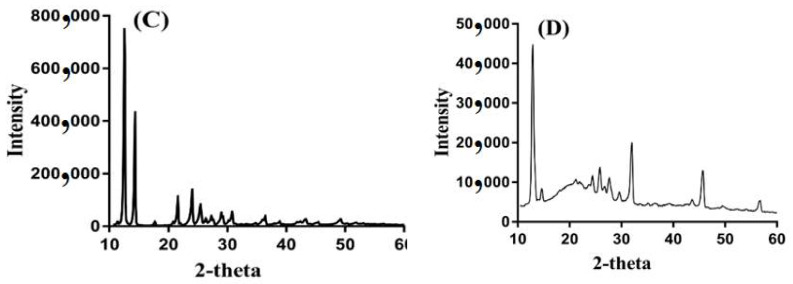
PXRD of (**A**) ASPA, (**B**) unloaded ASPA-pAMPS microgels (AFn-3), (**C**) TP, and (**D**) loaded ASPA-pAMPS microgels (AFn-3).

PXRD was carried out to analyze the crystallinity of the ASPA, unloaded ASPApAMPS microgels, TP, and drug-loaded ASPA-pAMPS microgels, respectively, as shown in Figure 8A–D. The PXRD of ASPA (Figure 8A) demonstrated prominent, high intensity crystalline peaks at 2θ = 22.80°, 24.30°, 26.20°, and 38.22°. The intensity of the characteristic peaks of ASPA disappeared/were reduced by unloaded ASPA-pAMPS microgels (Figure 8B), which revealed the successful polymerization of ASPA with AMPS and resulted in the development of ASPA-pAMPS microgels. Similarly, the high intensity crystalline peaks of TP were assigned at 2θ = 12.09°, 14.53°, 21.08°, 37.51° [46] (Figure 8C), respectively. Similarly, the crystallinity of the TP was reduced by the developed microgels as the high intensity crystalline peaks of the TP disappeared in the drug-loaded ASPA-pAMPS microgels (Figure 8D), which reveals that the developed system enhanced and sustained the delivery of the TP for a long period of time. The PXRD pattern of unloaded ASPA-pAMPS microgels and drug-loaded ASPA-pAMPS microgels indicated a slight difference in peak intensity due to the encapsulation of the drug by the fabricated system of microgels [29]. 

## 3. Conclusions

The characteristics of individual components were changed by the cross-linking of ASPA and AMPS and developed a new ASPA-pAMPS microgel carrier system by free radical polymerization technique. DSC and TGA demonstrated that the polymeric microgels were thermally stable due to the cross-linking and formation of different chemical bonds that enhanced the stability of the developed microgels. A hard surface with few pores of microgels was revealed by SEM. FTIR confirmed the development of ASPA-pAMPS microgels by polymerization reaction and the overlapping of AMPS on the backbone of ASPA. PXRD presented the reduction in crystallinity of the theophylline as the intensity of high crystalline peaks of the drug was reduced by polymeric microgels. pH-dependent swelling and percent drug release profiles were exhibited by designed microgels. High dynamic swelling and percent drug release were observed at pH 7.4 as compared to pH 1.2 due to the deprotonation of functional groups of ASPA and AMPS. Dynamic swelling, drug loading, and percent drug release were increased as the concentration of ASPA and AMPS was increased while they decreased with the enhancement in MBA concentration. Furthermore, a drug release study was performed for the commercial product, Theolin S.R tablets, at both pH 1.2 and 7.4. A rapid percent drug release (96%) from the commercial product was observed within the initial 6–8 h at pH 7.4, while almost the same percent drug release was observed at pH 1.2 within the initial 10 h. On the other hand, polymeric microgels sustained the high percent drug release for 24 h at pH 7.4, demonstrating sustained drug release behavior. Similarly, sol–gel fractions revealed an increase in the gel fraction with the increase in the composition of ASPA, AMPS, and MBA while showing a decrease in the sol fraction. Hence, due to the unique features of ASPA and AMPS that enabled the polymeric microgels to swell highly and release the high amount of drug at high pH values in a sustained manner, we can conclude that the current polymeric microgels are not limited to only sustaining the release of theophylline but can also be used for the sustained delivery of other drugs too, especially those experiencing stomach acidity problems.

## 4. Materials and Methods

### 4.1. Materials

Theophylline (TP) was obtained from Sigma-Aldrich (St. Louis, MI, USA). Poly(aspartic acid) (ASPA, MW: 133.10, purity = 99 plus%) was purchased from ACROS, NJ, USA. Moreover, 2-acrylamido-2-methylpropanesulfonic acid (AMPS) (purity = 98%) and N,N′-methylene bisacrylamide (MBA, purity = 97%) were procured from Alfa Aesar (Lancashire, UK). Similarly, ammonium peroxodisulfate (APS) (purity = 98%) and sodium hydrogen sulfite (SHS) (purity = 58.5%) were acquired from Showa (Tokyo, Japan) and Avantor Performance Materials (LLC, Radnor, Italy), respectively.

### 4.2. Synthesis of Microgels

Various compositions of polymer ASPA, monomer AMPS, and cross-linker MBA were employed at a constant concentration of initiators APS and SHS for the development of aspartic acid-co-poly (2-acrylamido-2-methylpropanesulfonic acid) (ASPA-pAMPS) microgels, as shown in Table 3. The specific amounts of ASPA, AMPS, MBA, and APS/SHS were taken separately and dissolved in their respective solvents. ASPA is soluble in water; hence, the required quantity of ASPA was dissolved in deionized distilled water. Similarly, AMPS and APS/SHS are completely soluble in water, so they were dissolved in a specific volume of deionized distilled water, respectively. MBA is not completely soluble in water, therefore a mixture of water and ethanol was used and stirred at 50 °C with 50 rpm. Initially, the APS/SHS solution was added into the AMPS solution, stirred for 5 min, then the mixture was poured into the polymer solution and stirred for 20 min. Finally, the MBA solution was added dropwise into the above mixture with constant stirring. After 5 min, a translucent solution was formed and purged by nitrogen gas in order to remove dissolved oxygen from the solution. The translucent solution was transferred into glass molds and these were placed in a water bath at 65 °C for 2 h initially, and then the temperature was enhanced up to 70 °C for the next 5 h. The prepared gel was passed through a specific mesh number, 20, and the fine particles of gels were obtained. A mixture of water and ethanol was used for washing in order to remove any unreacted content attached to the surface of the gels. The prepared gels were placed at room temperature initially for 24 h, and then placed in a vacuum oven at 40 °C until complete dehydration. The dried particles of gels were passed again through a mesh number of 625, and microgel particles were obtained. The prepared microgels were then evaluated for further experiments.

### 4.3. Dynamic Swelling

A swelling study was performed in HCl buffer of pH 1.2 and phosphate buffer of pH 7.4 at 37 °C for all formulations of APSA-pAMPS microgels. In total, 100 mg of microgels was enclosed in dialysis bags (MW; 12,000—14,000) and then immersed in respective buffer solutions. The dialysis bags were removed from the solution after a specific interval of time, blotted with filter paper to remove excess of water, weighed on a weighing balance, and then immersed again in respective pH buffer solutions. This process was continued until no further increase was observed in the weight of microgels [47]. The dynamic was calculated by the given equation:

(1) $$q=\frac{{\;D}_{2}}{{\;D\;}_{1}}\;$$

where q = dynamic swelling, D_1_ = initial weight of microgels before swelling, and D_2_ = final weight of microgels after swelling at time t.

### 4.4. Drug Loading

A drug loading study was conducted by diffusion and absorption method for fabricated microgels. A precise quantity of microgels was immersed into 2% drug solution of phosphate buffer pH 7.4, sonicated (Ultrasonic cleaner DC 400H) for 25 min, and then placed in an open area for 24 h at room temperature so that the maximum amount of the drug could be loaded by the microgels. The suspension was filtered by a membrane filter to remove the unloaded drug. After that, the suspension was lyophilized for 24 h to remove the entrapped solvent [48].

An extraction method was used for calculating the drug loaded by the developed microgels. An accurate weighed amount of drug loaded ASPA-pAMPS microgels was immersed in 100 mL phosphate buffer solution of pH 7.4 and stirred until the entire loaded drug was released. The suspension was filtered by a membrane filter and then analyzed on a UV-vis spectrophotometer (U-5100, 3J2-0014, Tokyo, Japan) at λ max 272 nm and the drug contents were evaluated.

### 4.5. Sol–Gel Analysis

Sol is the un-cross-linked soluble part of microgels, while gel is the cross-linked insoluble part of microgels. Sol–gel analysis was carried out for the purpose of knowing the sol and gel fractions of the ASPA-pAMPS microgels. Therefore, a specific amount of microgel (S_1_) was taken and added into the round bottom flask containing deionized distilled water. A condenser was fitted with the round bottom flask. The Soxhlet extraction process was carried out for 12 h. After that, the microgels were collected and allowed to dry in a vacuum oven at 40 °C. The dried microgels were weighed (S_2_) again [49]. The given equations were used for the analysis of sol–gel fractions:

(2) $$\mathrm{Sol}\;\mathrm{fraction}\;\%=\frac{{\;S}_{1}-{\;S}_{2}}{{\;S}_{2}}\times 100$$

where S_1_ = initial weight of microgels, and S_2_ = final weight of microgels.

(3) Gel fraction=100−Sol fraction

### 4.6. In Vitro Drug Release Study

An in vitro drug release study was performed with a calibrated 8 station dissolution apparatus (USP dissolution apparatus-II, Sr8plus dissolution test station, Hanson Research, Chatsworth, CA, USA) equipped with peddles at both pH 1.2 and 7.4, respectively. In total, 500 mL of both pH solutions was used for dissolution. Weighed quantities of drug-loaded microgels were placed in dialysis bags (MW; 12,000–14,000) and immersed in the respective medium at 37 ± 0.5 °C with 50 rpm. Similarly, commercially available tablets, Theolin S.R (250 mg, PeiLi Pharmaceutical IND. Co., Ltd.), were immersed in the respective buffer solutions of pH 1.2 and 7.4, under the same conditions. Aliquot of 5 mL was taken after a regular interval of time and a medium of the same quantity was added back to maintain the sink condition constant throughout the dissolution experiment. The collected aliquots were analyzed on a UV-vis spectrophotometer (U-5100, 3J2-0014, Tokyo, Japan) at λ max 272 nm in triplicate [43]. In order to deduce the release mechanism of TP from ASPA-pAMPS microgels, zero order, first order, Korsmeyer–Peppas, and Higuchi kinetic models were applied for all formulations of the developed microgels [36]. The following equations were used for the calculation of various kinetic modeling:

zero order kinetics Ft = K_0_t (4)

where Ft = fraction of drug released at time t, and K_0_ = zero order release constant;

first order kinetics ln (1−F) = K_1_t (5)

where F = fraction of drug released at time t, and K_1_= first order release constant;

Higuchi model F = K_2_t^1/2^ (6)

where F = fraction of drug released at time t, and K_2_= Higuchi constant;

Korsmeyer–Peppas model Mt/M_∞_= K_3_t^n^ (7)

where Mt/M∞ = fraction of drug release at time t, n = release exponent, and

K_3_ = rate constant.

### 4.7. Data Analysis

Statistical analysis was performed by using the computer program SPSS Statistic software 22.0 (IBM Corp, Armonk, NY, USA). By using the Student’s *t*-test, the differences between tests were tested and were considered statistically significant because the *p*-value was <0.05.

### 4.8. Differential Scanning Calorimetry (DSC) Analysis

DSC (PerkinElmer DSC 4000) was performed for ASPA, AMPS, unloaded ASPA-pAMPS microgels, TP, and drug-loaded ASPA-pAMPS microgels, respectively. A specific amount of all samples (0.5–5 mg) was kept in an aluminum pan. Nitrogen flow, heating rate, and temperature were kept constant throughout the study (nitrogen flow, 20 mL/min; heating rate, 20 °C/min; and temperature, 50–400 °C) [50].

### 4.9. Thermogravimetric Analysis (TGA) 

TGA (PerkinElmer Simultaneous Thermal Analyzer STA 8000) was conducted for ASPA, AMPS, and ASPA-pAMPS microgels. For TGA analysis, samples of polymer, monomer, and formulation were taken within a range of 0.5–5 mg and placed in an open pan connected to a microbalance. The heating rate was maintained at 20 °C/min. Similarly, the heat was kept at 40–600 °C under dry nitrogen throughout the experiment [50].

### 4.10. Surface Morphology and Particle Size Analysis

The surface morphology of ASPA-pAMPS microgels was analyzed by scanning electron microscopy (SEM) (JSM-5300). The sample of developed microgels was fixed on a double-adhesive tape stuck to an aluminum stub. Gold sputter was used for the coating of gold on stubs and performed beneath an argon atmosphere. Scanning of coated samples was performed randomly and, by the help of photomicrographs, surface morphology was evaluated [51]. For particle size analysis, microgel particles were dispersed in acetone and a suspension was formed, which was then used for the analysis of particle size by using dynamic light scattering (DLS) method (ELSZ-2000 particle size analyzer, Otsuka Electronics, Otsuka, Japan) [52].

### 4.11. Fourier Transform Infrared Spectroscopy (FTIR) Analysis

FTIR spectra of ASPA, AMPS, unloaded ASPA-pAMPS microgels, TP, and drug-loaded ASPA-pAMPS microgels were performed to know (i) the structural arrangement of the microgels’ contents individually and also in the polymeric network of microgels, and (ii) the interaction of drugs with the microgels’ contents. ATR (Attenuated Total Reflectance) mode was used for spectra analysis. Hence, all the samples were evaluated and analyzed by NICOLET 380 FTIR within the spectra range of 4000–500 cm^−1^ [53]. The number of scans and resolution were kept at 8 and 4 cm^−1^ throughout the study, respectively.

### 4.12. Powder X-ray Diffractometry (PXRD) Analysis

PXRD pattern of ASPA, AMPS, unloaded ASPA-pAMPS microgels, TP, and drug-loaded ASPAp-AMP microgels was carried out by XRD-6000 SHIMADZU X-ray DIFFRACTOMETER. Hence, dried powder samples of 500 mg were taken and griped by a plastic sample holder, whereas the surface of the samples was leveled by a glass slide. Theta (θ) was kept between 10–60° at a rate of 2° 2θ/min at room temperature [54].

## Figures and Tables

**Table 1:** Sol–gel analysis and % drug loading of ASPA-pAMPS microgels.

Formulation Code	Sol Fraction (%)	Gel Fraction (%)	Drug-Loaded (% *w*/*w*)
AFn-1	22.80 ± 0.07	77.20 ± 0.09	80.12 ± 0.51
AFn-2	16.89 ± 0.11	83.11 ± 0.04	84.44 ± 0.25
AFn-3	14.75 ± 0.09	85.25 ± 0.08	87.24 ± 0.31
AFn-4	26.80 ± 0.08	73.20 ± 0.10	78.62 ± 0.11
AFn-5	21.50 ± 0.13	78.50 ± 0.13	82.86 ± 0.44
AFn-6	15.70 ± 0.14	84.30 ± 0.12	83.80 ± 0.16
AFn-7	26.78 ± 0.10	73.22 ± 0.09	78.02 ± 0.15
AFn-8	22.88 ± 0.09	77.12 ± 0.11	74.88 ± 0.27
AFn-9	17.19 ± 0.12	82.81 ± 0.10	71.12 ± 0.34

**Table 2:** Kinetic modeling release of TP from ASPA-pAMPS microgels.

F. Code	Zero Order r^2^	First Order r^2^	Higuchi r^2^	Korsmeyer–Peppas	
				r^2^	n
AFn-1	0.8325	0.9655	0.9400	0.8787	0.7340
AFn-2	0.8599	0.9876	0.9787	0.9562	0.5651
AFn-3	0.8606	0.9918	0.9619	0.9160	0.5363
AFn-4	0.9180	0.9840	0.9742	0.9795	0.8526
AFn-5	0.8619	0.9753	0.9587	0.9445	0.5355
AFn-6	0.9089	0.9923	0.9811	0.9765	0.4886
AFn-7	0.7425	0.9647	0.8814	0.8971	0.4962
AFn-8	0.7845	0.9549	0.9125	0.8874	0.6015
AFn-9	0.8494	0.9324	0.9555	0.9294	0.6662

**Table 3:** Feed ratio scheme for formulation of ASPA-pAMPS microgels.

Formulation Code	Polymer (ASPA) g/20 g	Monomer (AMPS) g/20 g	Initiator (APS/SHS) g/20 g	Cross-Linker (MBA) g/20 g
AFn-1	0.100	3.0	0.1/0.1	1.0
AFn-2	0.170	3.0	0.1/0.1	1.0
AFn-3	0.240	3.0	0.1/0.1	1.0
AFn-4	0.100	4.0	0.1/0.1	1.0
AFn-5	0.100	5.0	0.1/0.1	1.0
AFn-6	0.100	6.0	0.1/0.1	1.0
AFn-7	0.100	3.0	0.1/0.1	1.1
AFn-8	0.100	3.0	0.1/0.1	1.2
AFn-9	0.100	3.0	0.1/0.1	1.3
